# Raman Spectral Characterization of Urine for Rapid Diagnosis of Acute Kidney Injury

**DOI:** 10.3390/jcm11164829

**Published:** 2022-08-18

**Authors:** Ming-Jer Jeng, Mukta Sharma, Cheng-Chia Lee, Yu-Sheng Lu, Chia-Lung Tsai, Chih-Hsiang Chang, Shao-Wei Chen, Ray-Ming Lin, Liann-Be Chang

**Affiliations:** 1 Department of Electronic Engineering, Chang Gung University, Taoyuan 333, Taiwan; 2 Kidney Research Center, Department of Nephrology, Change Gung Memorial Hospital, Linkou Branch, Taoyuan 244, Taiwan; 3 Green Technology Research Center, Chang Gung University, Guishan, Taoyuan 333, Taiwan; 4 Department of Cardiothoracic and Vascular Surgery, Chang Gung Memorial Hospital, Linkou Branch, Taoyuan 244, Taiwan

**Keywords:** acute kidney injury, Raman spectroscopy, partial least squares, linear discriminant analysis, urine

## Abstract

Acute kidney injury (AKI) is a common syndrome characterized by various etiologies and pathophysiologic processes that deteriorate kidney function. The aim of this study is to identify potential biomarkers in the urine of non-acute kidney injury (non-AKI) and AKI patients through Raman spectroscopy (RS) to predict the advancement in complications and kidney failure. Selected spectral regions containing prominent peaks of renal biomarkers were subjected to partial least squares linear discriminant analysis (PLS-LDA). This discriminant analysis classified the AKI patients from non-AKI subjects with a sensitivity and specificity of 97% and 100%, respectively. In this study, the RS measurements of urine specimens demonstrated that AKI had significantly higher nitrogenous compounds, porphyrin, tryptophan and neopterin when compared with non-AKI. This study’s specific spectral information can be used to design an in vivo RS approach for the detection of AKI diseases.

## 1. Introduction

Acute kidney injury (AKI) is one of the most non-infectious and potentially treatable but serious clinical diseases that affects between 5% and 13% of the general population across the world [1]. Although there have been improvements in dialysis and kidney transplantation techniques over the past three decades, high mortality and morbidity rates remain. Worldwide, over 750 million patients are affected by kidney disease, and an estimated 2 million patients per year are affected by kidney failure [2].

This disease usually remains asymptomatic until the advanced stages. The diagnosis of AKI relies on blood tests (creatinine, blood urea), serum creatinine levels, an abrupt decrease in glomerular filtration, urine output tests, and, sometimes, several urinary markers of kidney injury supplemented with time-consuming kidney biopsies that have low sensitivity and specificity [3]. The late diagnosis of AKI is associated with disease advancement, which requires expensive treatments and results in delayed or impaired recovery and an increased mortality rate [4].

This necessitates the identification of a simple, quick and non-destructive diagnosis technique for the detection of AKI. When kidney disease is present, the chemical composition and constitution of identified materials in urine change [5]. Remarkable urine biomarker investigations of the detection of chronic renal disease have taken place in research settings.

Urine is of particular relevance since it allows for a painless and non-invasive sample collection process and because it includes over 3000 metabolites or metabolic species that can be exploited for diagnostic purposes [6]. The most common organic chemicals found in urine are urea, creatinine, hippuric acid and citric acid.

Raman spectroscopy (RS) is the most widely used optical technique for providing specific fingerprint-type information on molecules. It uses laser light to explore a molecule’s vibrational modes (which are sensitive to its chemical bonds) through an inelastic scattering of incident radiation by the polarization of molecules [7]. Urine analysis using RS offers several advantages over standard chemical methods, such as there being no need for dilution and reagents, shorter evaluation times, more comprehensive information and a small volume of samples [8].

In the last few years, spectroscopic approaches combined with multivariate statistical analyses have provided new approaches to the early screening of different types of cancer [9,10,11,12,13].These approaches help to monitor minor spectrum deviations that can be associated with specific diseases. Furthermore, they provide quick and non-destructive analyses without sample preparation, making it ideal for screening tests. There are numerous examples of biofluid-based RS being used to successfully screen for various cancer types in the literature [14,15,16,17,18,19,20,21].

This study aims to evaluate Raman spectra in the fingerprint region (500–1800 cm${}^{-1}$) of urinalysis in urine, providing a tool for differentiating non-AKI and AKI patients for the future control of kidney disease. We developed a quantitative model using partial least squares linear discriminant analysis (PLS-LDA) that can be applied to the RS of urine to evaluate the concentrations of all significant metabolites in the body that can be useful for AKI diagnosis.

## 2. Methods and Materials

### 2.1. Patients and Samples

The Chang Gung Memorial Hospital’s Institutional Review Board (IRB) approved this study through IRB No: 103-1993B and 202001691B0. Between June 2014 and July 2017, this study was conducted in the heart surgery intensive care unit (ICU) of a tertiary care referral center in Taiwan. The clinical data and pathological reports were collected in the Chang Gung Memorial Hospital’s Department of Nephrology. In this study, we used 200 urine samples. The mean age of the patients was 61.0 ± 14.8 years, and 66.8% of the patients were male.

The baseline mean serum creatinine was 1.1 ± 0.7 mg/dL. The most common cardiac surgery was coronary artery bypass graft (CABG) in 75 patients (37.5%), followed by heart valve surgery in 74 (37%), aorta surgery in 33 (16.5%) and CABG combined with valve surgery in 11 (5.5%). Postoperative AKI occurred in 80 patients (40%). Among these, 42 patients were diagnosed with AKI stage 1 (52.5%), 20 with AKI stage 2 (25%) and 19 with AKI stage 3 (23.8%).

The enrolled participants were admitted to the ICU immediately after cardiac surgery, and they provided written, informed consent for the collection of urine specimens. Patients on dialysis who were younger than 20 years old, had an estimated glomerular filtration rate of less than 30 mL/min/1.73 m${}^{2}$, had any prior organ transplantation, or had anuria immediately after surgery were excluded. Fresh urine samples were collected in sterile non-heparinized tubes during the first 4 h after surgery, and they were then centrifuged at 5000× *g* for 30 min at 4 °C to remove cells and debris.

The urine was dispensed into a centrifuge tube and stored in a freezer at −80 °C to prevent altering their morphology until use and was thawed to room temperature before measurements. To determine the predictive ability of RS for differentiating AKI and non-AKI, the primary outcome was the development of AKI within 7 days after cardiac surgery.

We defined the development of AKI using serum creatinine-based criteria of the Kidney Disease Improving Global Outcomes (KDIGO) Clinical Practice Guidelines for AKI. The current study included 200 consecutive patients who were all undergoing cardiac surgery. Thirty µL of urine samples were collected from each sample. Five spectra of each urine sample were obtained at different locations. Thus, 1000 spectra were obtained from the two types of urine samples.

### 2.2. Raman Spectral Data Acquisition

The Micro Raman Identify (MRI) system (ProTrusTech Co., Ltd., Tainan, Taiwan) was used, which has a laser with an excitation wavelength of 532 nm and a laser power of 126 mW. The integration and acquisition times were 5 and 15 s, respectively, and the average value of the spectrum was 3. This spectrometer provides a spectral resolution of 1 cm${}^{-1}$. Before undertaking the analyses, the specimens were taken out of the refrigerator and defrosted to room temperature for about an hour, after which 30 µL was pipetted onto aluminum foil. The RS was then used to record the Raman spectra of the urine.

### 2.3. Pre-Processing of the Spectra

MATLAB (R2018a, MathWorks, Natick, MA, USA) was used to process and analyze the data. To reduce interference, a Savitzky–Golay filter with an order of 3 was employed to smooth the recorded spectra. Then, after the baseline correction, normalization was performed to eliminate data redundancy.

### 2.4. Data Analysis

The spectral preprocessed data contained a set of 965 intensity variables from 500 cm${}^{-1}$ to 1800 cm${}^{-1}$. The classification was performed using linear discriminant analysis (LDA). To improve the diagnostic capability of the LDA classifier, it was necessary to reduce the dimensions of the urine’s Raman spectral data. Partial least squares (PLS) was used to extract features from the pre-processed Raman spectra of urine. The mean-normalized spectrum was analyzed using PLS-LDA, which is a multivariate statistical method.

The primary reason for using this multivariate statistical model is because the PLS method removes redundancy and noise in high-dimension datasets and because it allows the dimension reduction features to be used as the input for the LDA algorithm [22]. The PLS model was used for estimating the concentrations of renal function markers, including creatinine, urea, uric acid and others, along with the corresponding Raman spectra. In PLS discriminant analysis, the latent variables (LVs) are rotated to achieve maximum group separation. As a result, the LVs assess diagnostically important changes rather than significant variations in the dataset [18].

The LDA classifier was used to investigate the boundary between classes and classification probability [23]. To evaluate the classified results, the classifier models were optimized using a training dataset, and their performance was evaluated using a test dataset. The first nine components had a strong correlation coefficient of 0.84 between the X and Y scores in the PLS model. The PLS model’s output was fed into the LDA classifier, and the analysis broadly categorized the patients into either the non-AKI group or the AKI group. Five spectra were collected from the entire sample area that was measured for each sample measurement, and the mean of these five spectra was employed in the further data analysis.

To generate a three-dimensional scatter plot of PLS, the PLS components 1, 2 and 3 were used. A receiver operating characteristic (ROC) curve was used to visualize the classification of non-AKI and AKI patients and was drawn by plotting the sensitivity (true positive rate) against the 1-specificity (false positive rate).

### 2.5. Validation of Model Using a Testing Data Set

We divided our data set randomly into training and testing data sets. The training dataset consisted of 140 urine samples (84 non-AKI and 56 AKI patients), whereas the testing dataset consisted of 60 samples (36 non-AKI and 24 AKI patients), as detailed in Table 1. The spectra of 140 urine samples were used to train the classification model. The testing dataset was used to evaluate the model.

## 3. Results and Discussion

A total of 200 urine samples were measured using RS. Of these 200 samples, 140 samples were used to develop the AKI detection algorithm or classification model using the training dataset. The remaining 60 samples were used to validate the model using the testing dataset. The samples consisted of urine from 120 non-AKI and 80 AKI patients. For all AKI patients, there were 42 in the first stage, 19 in the second stage and 19 in the third stage.

### 3.1. Spectral Analysis

**Figure 1:**
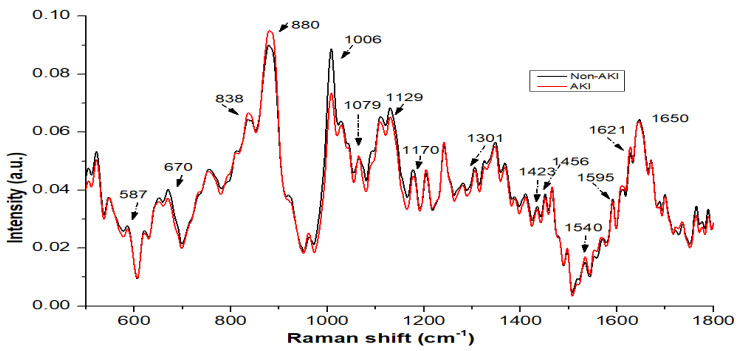
The mean Raman spectrum from 120 non-AKI and 80 AKI patients.

Figure 1 represents averaged, baselined and vector normalized urine spectra for the 120 non-AKI and 80 AKI patients in the wave-number region of 500–1800 cm${}^{-1}$. In our study, we observed that the mean spectra of these two groups were relatively similar, and the major contributions comprised urea, creatinine, porphyrin, nitrogenous compounds, hydroxybutyrate, tryptophan, CH${}_{2}$ bending mode and uric acid. More intense peaks of nitrogenous compounds (1,079,880 cm${}^{-1}$), porphyrin (1621 cm${}^{-1}$), tryptophan (838 cm${}^{-1}$) and neopterin (1540 cm${}^{-1}$) were observed in the AKI spectra than in the non-AKI spectra with normal kidney function.

However, prominent peaks of urea (587, 1006 and 1170 cm${}^{-1}$), creatinine (670 and 1423 cm${}^{-1}$), CH${}_{2}$ bending (1301 cm${}^{-1}$), hydroxybutyrate (1456 cm${}^{-1}$) and uric acid (1423, 1595 and 1650 cm${}^{-1}$) were seen in the non-AKI urine samples. There was also a sharp peak of porphyrin (1621 cm${}^{-1}$) in the AKI urine samples, which was possibly due to the effect of a metabolic disorder that causes an enzyme deficiency [24]. The nitrogenous compounds, which were mostly observed as ethanolamine bands at 880 and 1079 cm${}^{-1}$, were most likely created by the urea cycle’s amino acid metabolism, and higher concentrations were found in the urine of chronic kidney disease patients [25].

Urea and creatinine are important components of urine that can provide essential information about the health of the kidney and can assist with the detection of initial renal disorders [26,27]. In kidney disease, the metabolic capacity of the kidney is reduced, resulting in low signals of these substances (urea and creatinine) as shown in Figure 1. Tryptophan is a type of amino acid, and it was observed that it was elevated in the AKI patients compared with that of the non-AKI group. There was a sharp and intense peak at 1456 cm${}^{-1}$ in each group’s urine samples, which was present due to hydroxybutyrate assignment, and this decreased during kidney disorder or disease as shown in Figure 1.

The vibration of neopterin at 1540 cm${}^{-1}$ was observed both in the AKI and non-AKI subjects but had a higher intensity in the AKI patients than the non-AKI subjects. Assignment of uric acid (a metabolite of purine) at 1595 cm${}^{-1}$ was also observed in both groups, with minor differences in intensity. There were two small and sharp peaks of creatinine at 673 and 1423 cm${}^{-1}$ [17], with higher intensity in the control subjects.

**Figure 2:**
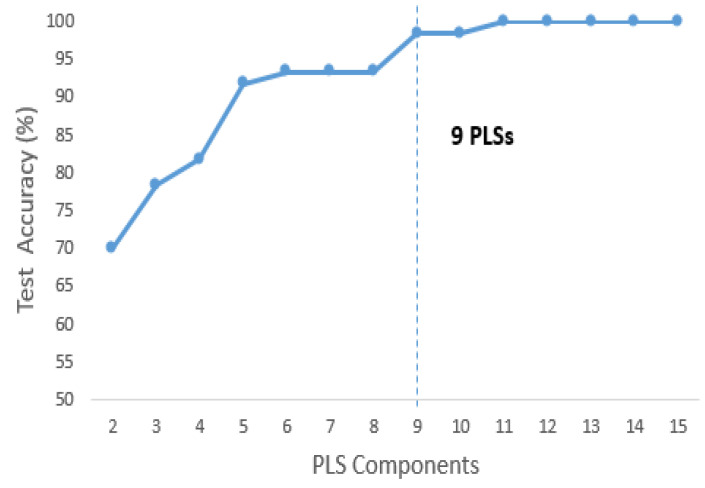
Accuracy rates vs. the number of PLS components in the urine sample dataset.

**Figure 3:**
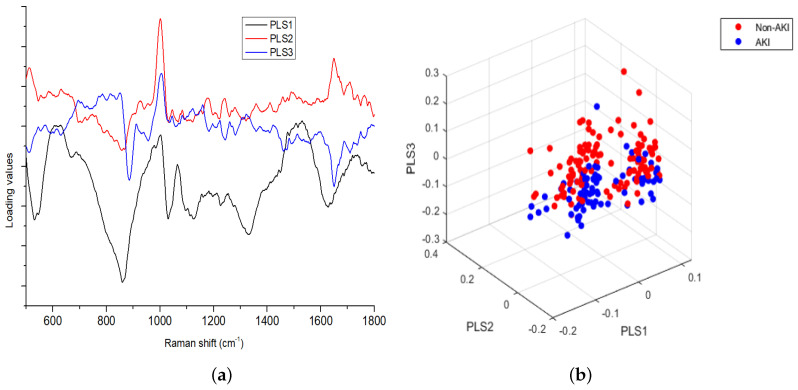
PLS (**a**) three loading factors and (**b**) 3D scatter plot.

The classification accuracy of the model improved with the number of components and tended to stay constant after a specific value had been reached, which is justified in Figure 2. Figure 3a illustrates the loading of the three main PLS components or LVs for the Raman spectral dataset. The loadings of PLS2 and PLS3 contained the following specific Raman peaks for uric acid, urea and creatine: 1006 cm${}^{-1}$, 1079 cm${}^{-1}$, 1170 cm${}^{-1}$, 1423 cm${}^{-1}$ and 1650 cm${}^{-1}$. The loading on the Raman peaks captured by the first PLS component was mainly associated with urea and nitrogenous compounds as demonstrated by the peaks at 1079 cm${}^{-1}$ and 1006 cm${}^{-1}$.

The first nine PLSs were selected to build classification models; however, the first three contributing components were extracted to create the scatter plot shown in Figure 3b.

### 3.2. Evaluation of Model Using Testing Set

To avoid over-fitting, 70% of the data was used in the training dataset, and 30% was used in the testing dataset. To identify the AKI, k-fold cross-validation (k = 5) was employed with the LDA classifiers. The data were separated into k equal folds. The algorithm was then trained to work with the k-5 folds with the remaining fold being used as the testing dataset. The results were averaged across k folds. This test set comprised 300 Raman spectra obtained from 60 urine samples, 24 of which were AKI patients, and 36 of which were non-AKI subjects. A test dataset for a PLS-LDA model was performed for the PLS components from 1 to 15 (Figure 2).

**Figure 4:**
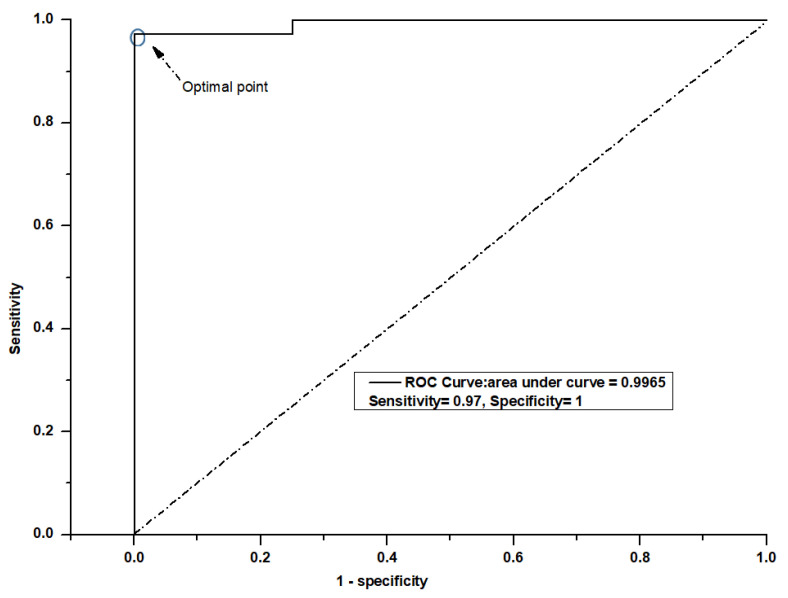
ROC curve of the classification results for the PLS-LDA model.

Then, by determining a saturation point for accuracy as a function of the PLS number, an optimal number of PLS components was selected. These significant PLS components were used as input for the LDA. The PLS-LDA model correctly classified 36 of the 36 non-AKI subjects and 23 of the 24 AKI patients with the urine spectra. This model had an accuracy of 98.5%, a sensitivity of 97% and a specificity of 100% for classification (Table 2). The ROC curves for the PLS–LDA model are presented in Figure 4, which shows that the maximum value of the area under the ROC curve (AUC) was 99.7.

We demonstrated that RS is a useful tool for diagnosing AKI from urine samples. According to KDIGO, AKI is classified into three severity categories (AKI stage 1, 2 and 3) based on serum creatinine levels, reduced urine output and the necessity for renal replacement therapy. However, a limited number of patients who suffer from these three stages were included. As a result, we did not attempt to distinguish the stage of kidney disease in this investigation. Urine is a product of the kidney and can be collected non-invasively and is considered a promising source of information for renal biomarker studies.

Using RS to identifythe chemical constituents present in urine, such as nitrogen compounds, urea, creatinine and uric acid, as well as their rate of excretion was proposed [28]. The kidney is capable of filtering 90% of the urea that is metabolized. Urea measurement has previously been used to assess nutritional factors from urine, including insufficient protein consumption and food deprivation.

Although it is not clearly defined for any kidney-related disorders, it is still responsive to early-stage kidney problems [27]. Creatinine is a product of muscle metabolism that is related to the body’s muscle mass. As daily metabolic creatinine is generally constant in healthy people, creatinine detection is one of the most popular methods for diagnosing kidney function [26].

A PLS–LDA model was used to analyze the statistical efficiency of this tool. The PLS data were calculated for non-AKI and AKI patients in the fingerprint region 500–1800 cm${}^{-1}$. The spectral data were analyzed according to the first nine PLS components. The PLS–LDA model is preferable to spectral data and is the most effective multivariate statistical technique [29]. A ROC curve was used to investigate this model’s clinical potential, and the AUC provided an estimation of the sensitivity and specificity to any degree of significance.

Most of the biomolecular information from urine is crucial for distinguishing AKI patients from the non-AKI patients, according to this model. In this study, all of the urine samples were obtained from heart surgery patients, and we attempted to acquire a higher sensitivity and specificity using the PLS-LDA classification model. In this study, we found that the Raman spectra of AKI urine specimens demonstrated that it had significantly higher nitrogenous compounds, porphyrin, tryptophan and neopterin than non-AKI, while the Raman spectra of non-AKI urine specimens demonstrated that it had significantly higher urea (587, 1006 and 1170 cm${}^{-1}$), creatinine (670 and 1423 cm${}^{-1}$), CH2 bending (1301 cm${}^{-1}$), hydroxybutyrate (1456 cm${}^{-1}$) and uric acid (1423, 1595 and 1650 cm${}^{-1}$) compared with AKI.

AKI patients had a lower amount of urea, creatinine and uric acid in comparison to non-AKI Patients. In this study, the difference between the AKI and non-AKI groups is minor. The reason may be that most AKI patients belong to the first stage, which is producing the small variation in both types of spectra.

Although the medical field has currently made considerable progress, new diseases and complications still pose a great threat to human life. In comparison to new diseases, we are more aware of the various complications that accompany AKI; however, sometimes treatment is delayed because of a failure to detect it in time or because of errors in the diagnosis, resulting in sequelae or even death of the patient. Using RS, the biomolecules present in urine can be analyzed rapidly, which may reduce this occurrence.

## 4. Conclusions

This study presents a non-invasive, rapid and simple method for diagnosing the condition of kidney disease in patients. Changes in the distribution and conformation of urinary metabolites, such as creatinine, urea, uric acid, porphyrin, nitrogenous compounds, hydroxybutyrate and tryptophan, may be responsible for the spectral differences observed between non-AKI subjects’ and AKI patients’ urine. PLS-LDA was used to analyze the spectral data with an accuracy of 98.5%. However, this study’s sample size was limited. In the future, this research should be extended by having a larger sample in order to support this preliminary finding and further explore the potential of RS combined with advanced machine-learning techniques to facilitate the early diagnosis of AKI. 

## Figures and Tables

**Table 1:** Classification of all tested urine samples under Raman spectroscopy: Training set and test set.

Data Set	Non-AKI	AKI	Total
Training	84	56	140
Testing	36	24	60

**Table 2:** Test set misclassifications and performance tables of the PLS-LDA model.

Dataset	Confusion Table			Performance Parameters		
**PLS-LDA**	**Non-AKI**	**AKI**	**Total**	**Accuracy (%)**	**Sensitivity (%)**	**Specificity (%)**
Non-AKI	36	0	36	98.5	97	100
AKI	1	23	24			

## Data Availability

Not applicable.
